# Evaluation of the multivalent immune protective effects of the *Vibrio fluvialis* outer membrane protein VF17320, and its DNA and IgY antibody vaccines in fish

**DOI:** 10.3389/fvets.2025.1586258

**Published:** 2025-06-18

**Authors:** Huihui Xiao, Pan Cui, Jing Chen, Libing Meng, Xixian Che, Zijian Ma, Xiaoqing Wu, Juan Lu, Shujun Sun, Guoping Zhu, Yong Liu, Xiang Liu

**Affiliations:** ^1^Anhui Province Key Laboratory of Embryo Development and Reproductive Regulation, Anhui Province Key Laboratory of Environmental Hormone and Reproduction, Fuyang Normal University, Fuyang, China; ^2^Rural Revitalization Collaborative Technology Service Center of Anhui Province, Fuyang Normal University, Fuyang, China; ^3^Anhui Provincial Key Laboratory of Molecular Enzymology and Mechanism of Major Metabolic Diseases, Auhui Provincial Engineering Research Centre for Molecular Detection and Diagnostics, College of Life Sciences, Anhui Normal University, Wuhu, China

**Keywords:** *Vibrio fluvialis*, multivalent vaccine, protein vaccine, IgY vaccine, DNA vaccine

## Abstract

**Introduction:**

Vaccines exhibit characteristics such as no residue, low drug resistance, and environmental friendliness, and demonstrate application value in aquaculture.

**Methods and results:**

The protein, DNA, and egg yolk antibody (IgY) vaccines targeting the *Vibrio fluvialis* outer membrane protein VF17320 were immunized to fish (*Carassius auratus*) and subsequently challenged with *V. fluvialis* and *Aeromonas hydrophila*. The results indicated that the three vaccines enhanced the expression levels of immune-related factors of acid phosphatase (ACP), alkaline phosphatase (AKP) and lysozyme (LZM) (*p* < 0.01), provided immune protection against bacterial infections (*p* < 0.01), effectively reduced kidney bacterial counts (*p* < 0.01), and increased the phagocytic activity of white blood cells in *C. auratus* (*p* < 0.01). Furthermore, the three vaccines downregulated the expression of inflammation-related genes (*p* < 0.01) and antioxidant-related factors (*p* < 0.01) to mitigate the inflammatory and antioxidant response in *C. auratus*, respectively. Histopathology revealed that the three vaccines preserved the integrity of visceral tissue, and immunofluorescence demonstrated that the vaccines reduced apoptosis and DNA damage in the kidney cells induced by bacterial infection.

**Discussion:**

Overall, the three vaccines exhibit the potential to combat various bacterial infections and can serve as multivalent vaccine candidates for aquaculture.

## 1 Introduction

Aquaculture is one of the pillar industries of agriculture, with enormous economic benefits. Due to large-scale aquaculture, bacterial diseases constrain the development of the aquaculture industry. *Vibrio fluvialis* is a prevalent Gram-negative bacterium commonly found in soil and aquatic environments (1). This bacterium has a broad host range and is particularly prone to infecting commonly farmed fish species (2). Infected fish exhibit symptoms such as meningoencephalitis, skin lesions, and sepsis, which are associated with an extremely high fatality rate (3). Consequently, it has emerged as a significant fish pathogen, leading to substantial economic losses in freshwater aquaculture (4). Additionally, *V. fluvialis* is recognized as a zoonotic pathogen capable of infecting both animals and humans, where it can cause gastroenteritis or profuse watery diarrhea, and has been linked to enterocolitis in infants (5). Therefore, controlling infections caused by *V. fluvialis* has become a critical concern in the field of fish health aquaculture (6).

The prevention and control of bacterial pathogens mainly rely on antibiotics (7). However, the misuse of antibiotics can lead to significant issues, including antibiotic residues, the development of bacterial resistance, and environmental pollution (8). To enhance disease resistance in fish, feed is supplemented with vitamins, herbs, and probiotics to exert antibacterial effects (9, 10). Nevertheless, these approaches have limitations in effectively addressing pathogenic bacterial infections. Vaccines have garnered research interest due to their benefits, which include the avoidance of antibiotics, the absence of toxic side effects and residues (11, 12). In aquaculture, the vaccines commonly used in practical applications are attenuated live vaccines or inactivated vaccines, and the protein, DNA, and IgY antibody vaccines are mostly in the laboratory research stage. The protein vaccine does not contain nucleic acid and can induce the body to produce antibodies using a certain surface structural component (antigen), and has the advantages of high purity, strong targeting, good stability, and high safety (13). The recombinant DNA vaccines can introduce DNA fragments encoding specific antigens into the host cell, causing it to express the antigen that induces the effective immune response in the body. The DNA vaccine has the advantages of high stability, long-lasting immune response and low cost, while the disadvantages or potential dangers include possible integration with the host genome (14). IgY antibody vaccine is a biological preparation based on egg yolk immunoglobulin, which can be obtained from egg yolks by laying hens immunization, and can be used for the prevention and control of pathogens (15). Egg yolk IgY antibody can be produced on a large scale, with simple and economical preparation, and no drug residue, making them promising for application in aquaculture. It is necessary to utilize the characteristics of the protein, DNA, and IgY antibody vaccines to develop new vaccines for the prevention and control of pathogenic bacteria in aquaculture.

The outer membrane proteins (OMPs) are situated on the surface of bacteria, with its epitopes being readily accessible and easily recognized by the host immune defense system, thereby stimulating immune responses (16, 17). All Gram-negative bacteria contain surface-associated outer membrane proteins, many of which have been regarded as potential vaccine candidates (18). OMPs are crucial for maintaining the integrity and selective permeability of the bacterial membrane, and they play a significant role in various bacterial adaptive responses, including solute and ion uptake, iron acquisition, antimicrobial resistance, serum resistance, and resistance to bile salts. Additionally, some adhesins exhibit virulence properties (19). Previous studies have demonstrated that *Bordetella pertussis* OmpA can induce the production of specific serum antibodies in mice, providing a significant protective effect against challenges with *Pseudomonas aeruginosa* (20). Following the challenge of the *Vibrio alginolyticus* recombinant protein Lrp in pearl gentian grouper, Wan et al. reported that the immune protection rate in the Lrp group was increased to 60% (21). Additionally, the TonB-dependent copper receptor in *Acinetobacter baumannii* has been identified as a potential vaccine candidate (22). Howlader et al. developed a candidate subunit vaccine, termed ExlA/L-PaF/BECC/ME, and immunized older mice with it before infecting them with *Pseudomonas aeruginosa*. The results indicated that the mice immunized with ExlA/L-PaF/BECC/ME exhibited T cell-mediated adaptive responses, elicited the immune response against *P. aeruginosa*, and demonstrated reduced inflammatory responses (23). Consequently, this vaccine shows promise for protecting older adults from *P. aeruginosa* infection. Furthermore, the OMP VF17320 of *V. fluvialis*, located in the outermost layer of the bacteria, may be involved in signal transduction, energy metabolism, and cell integrity. However, the immune response and protective effects associated with *V. fluvialis* VF17320 remain unreported.

This study utilized the outer membrane protein VF17320 of *V. fluvialis* ATCC33809 as a starting point to investigate three types of vaccines: the VF17320 protein active immunity vaccine, the VF17320 DNA vaccine, and a passive immunity vaccine based on VF17320 IgY antibodies derived from eggs. *C. auratus* were immunized with these three vaccines and subsequently challenged with *V. fluvialis* and *A. hydrophila*. Various methods, including immune activity analysis, protection rate testing, assessments of anti-inflammatory and antioxidant effects, histopathological examination, and immunofluorescence (Supplementary Figure 1), were employed to evaluate the immune efficacy of the vaccines. This study provides a foundational basis for the development of vaccines in aquaculture.

## 2 Materials and methods

### 2.1 Strains and animals

The strains used in this study include *V. fluvialis* ATCC33809, *A. hydrophila* ATCC7966, and *Staphylococcus aureus* ATCC6538. Additionally, the outer membrane protein VF17320 (GenBank: AMF95190.1) vaccine, along with the DNA vaccine of VF17320, is deposited in the Microbiology Study Laboratory of Fuyang Normal University. Twenty-week-old Leghorn laying hens were procured from Chongqing Tengxin Biotechnology Co., Ltd. (Chongqing, China), and red crucian carp (20 ± 1.0 g) were obtained from Fuyang Aquaculture Co., Ltd. (Fuyang, China). All animal experiments were conducted in accordance with the Guide for the Care and Use of Laboratory Animals and received approval from the Institutional Animal Care and Use Committee of Fuyang Normal University, China (no. 2024-04).

### 2.2 Preparation of IgY antibodies

Each chicken was immunized with 200 μg of protein administered four times, with a 14-day interval between each immunization. Freund’s complete adjuvant was utilized for the initial immunization, while Freund’s incomplete adjuvant was employed for the subsequent booster immunizations. Eggs were collected for a duration of 40 days following the immunizations. The yolks from selected eggs were separated using a yolk separator, and an equal volume of phosphate-buffered saline (PBS) at pH 7.2 was added and mixed thoroughly. Subsequently, 3.5% powdered PEG6 000 was incorporated, and the mixture was shaken at 25°C at a speed of 100 r/min for 30 min. After centrifugation at 10000 r/min, the supernatant was filtered through filter paper. An additional 8.5% PEG6 000 was added to the filtrate, mixed thoroughly, and then placed in a shaker at 25°C, shaken at 100 r/min for another 30 min. Following a second centrifugation at 10000 r/min, the supernatant was discarded, and the precipitate was dissolved in 10 mL of PBS. A 12% PEG6000 solution was then added, and the mixture was shaken at 100 r/min for 30 min. After standing for 10 min, the mixture was centrifuged again, and the precipitate was dissolved in 2 mL of PBS. Finally, the solution was placed into a dialysis bag and dialyzed against PBS for 36 h at 4°C (24).

### 2.3 Western blotting

Western blotting was employed to assess the specificity of IgY antibodies. *V. fluvialis* was cultured overnight at 37°C. Following bacterial collection via centrifugation, 300 μL of SDS loading buffer was added, and the mixture was shaken and mixed thoroughly before being boiled for 5 min. The resulting solution was then applied to the SDS-PAGE gel for electrophoresis, followed by a transfer to the membrane at 4°C and 80 V for 60 min. After electrophoresis, the NC membrane was blocked overnight at 4°C using 5% skim milk. The membrane was washed three times with TBST (Tris-HCl-Tween 20) and then incubated at room temperature for 2 h with a gradient dilution of IgY antibody (1:400–1:102400, multiple dilutions). Following this, the membrane was washed three additional times with TBST and incubated with a secondary antibody (HRP-conjugated Affinipure Rabbit Anti-Chicken IgY (IgG) (H + L), 1:1000 dilution) at 37°C for 1 h. After three final washes with TBST, the membrane was developed using ECL luminescent solution (24).

### 2.4 *In vitro* interaction detection

In vitro interaction detection employs the ELISA test to identify the interaction between IgY antibodies or *C. auratus* serum and bacteria, and the experiment was repeated three times. Briefly, *V. fluvialis* is cultured to an optical density (OD600) of 1.0, after which the bacteria were collected via centrifugation. The bacterial suspension was then adjusted to an OD600 of 1.0 using physiological saline, and 200 μL of bacterial solution was added to each well of the enzyme plate. The plate was coated at 4°C overnight and subsequently washed three times with TBST. Following a blocking step with 5% skimmed milk powder at 37°C for 1.5 h, the cells were washed three times with Tris-Borate-Sodium Tween-20 (TBST) solution. Gradient dilutions of IgY antibody (1:100, 1:200, 1:400, 1:800, 1:1600, 1:3200) were added to the wells. Meanwhile, the *C. auratus* sera were obtained on 2 days after being immunized with IgY antibodies and challenged with bacteria in *C. auratus*. Then, gradient dilutions of the *C. auratus* sera (1:100, 1:200, 1:400, 1:800, 1:1600, 1:3200) were also added to the wells. Subsequently, the enzyme plate was incubated at 37°C for 1 h, followed by three washes with TBST. A secondary antibody (HRP-conjugated Affinipure Rabbit Anti-Chicken IgY (IgG) (H + L), diluted 1:1000 or rat anti-fish IgM, diluted 1:400) were added, and the incubation continues at 37°C for 1 h. The plate is washed three times with TBST, after which a chromogenic solution is added and incubated in the dark at 37°C for 10 min. Finally, a stop solution is added to terminate the reaction, and the absorbance at OD450 is immediately read using a microplate reader (25).

### 2.5 The expression, purification, and verify of VF17320 protein

The VF17320 protein (recombination into pET-32a plasmid) was expressed and purified as previously described (26). Briefly, the recombination VF17320 strain was cultured at 37°C overnight and transferred to 600 mL fresh LB medium until bacterial concentration reached 0.6 (*OD*600 nm), and the final concentration of 0.3 mmol/L Isopropyl-*β*-D-thiogalactoside (IPTG) was added and induced for 24 h at 20°C to express VF17320 protein. After centrifugation at 8000 r/min for 2 min, bacteria were disrupted with ultrasonic crushing, and Ni-NTA flow resin (Sigma, St. Louis, MO, United States) was used to purify VF17320. Then, sodium dodecyl sulfate polyacrylamide gel electrophoresis (SDS-PAGE) was used to identify the purity of VF17320 protein. Briefly, the purified VF17320 was boiled for 5 min after addition of sample loading buffer, and electrophoresed with constant voltage of 100 V for the resolving gels until the racking dye (bromophenol blue) reached the bottom of the gels. The protein bands were visualized by staining with Coomassie Brilliant Blue G-250.

The verify of VF17320 purification was analyzed using western blotting, as previously described (26). Briefly, the purified VF17320 was performed with SDS-PAGE gel electrophoresis, and the proteins were transferred to a nitrocellulose (NC) membrane at 80 V for 1 h. Skim milk (5%) was used to block the NC membrane for 2 h at room temperature. The VF17320 mouse antibody of different dilutions (1: 800, 1: 1600) was added to the NC membrane and incubated at 37°C for 1 h. The NC membrane was incubated with secondary goat anti-mouse antibodies (Sigma), and a dimethylaminoazobenzene (DAB) substrate system (Sigma) was employed to visualize the bands to verify purified VF17320.

### 2.6 Active and active cross-protection rates of VF17320 protein

To assess the challenge dose of the pathogenic bacteria, the lethal dose (LD_50_) value of *V. fluvialis* or *A. hydrophila* in *C. auratus* was determined using forty fish evenly distributed among four separate tanks, each group containing ten fish for every pathogenic bacterium. A negative control without challenging bacterium contained ten fish was also maintained. Briefly, the concentration of VF17320 was adjusted to 2 μg/g, with an immune volume of 25 μL per fish. Freund’s incomplete adjuvant and VF17320 protein (50 μg) were combined and subsequently injected into the abdominal cavity of *C. auratus*. Two immunizations were administered on days 1 and 14. Seven days following the completion of immunization, the concentrations of *V. fluvialis* (4 × 10^8^, 6 × 10^8^, 8 × 10^8^, and 1 × 10^9^ CFU) or *A. hydrophila* (2 × 10^8^, 4 × 10^8^, 6 × 10^8^, and 8 × 10^8^ CFU) were intraperitoneally challenged to *C. auratus*, and mortality was observed for up to one week, respectively. The LD_50_ value of bacteria was calculated for the subsequent bacterial challenge test of vaccine protection rate (25).

The immune protective rate of VF17320 protein was evaluated by *C. auratus* immunizing VF17320 and challenging with *V. fluvialis* or *A. hydrophila* according to the methods of Liu et al. (25). Briefly, the concentration of VF17320 protein was adjusted to 2 μg/g, with an immune volume of 25 μL per fish. Freund’s incomplete adjuvant and VF17320 (50 μg) were combined and subsequently injected into the abdominal cavity of *C. auratus*. For the *V. fluvialis* challenging experiment, the *C. auratus* (20 ± 1.0 g) were divided into a control group (receiving physiological saline) and VF17320 group, with 15 fish in each group. Meanwhile, the fish used for the *A. hydrophila* challenging experiment were treated the same. Two immunizations were administered on days 1 and 14. Seven days following the completion of immunization, the concentrations of challenge *V. fluvialis* and *A. hydrophila* were determined to be 8 × 10^8^ CFU and 4.0 × 10^8^ CFU as preliminary LD_50_ determination experiment of fish bacterial challenge, respectively. Observations and recordings were conducted over a period of 14 days. The protection rate (RPS) was calculated using the formula: RPS (%) = (1−[% mortality rate of experimental group/ % mortality rate of control group]) × 100. SPSS 19.0 software was utilized to analyze the significant differences between the experimental and control groups (25).

### 2.7 Passive and passive cross-protection rates of VF17320 IgY antibody

For the *V. fluvialis* challenging experiment, the *C. auratus* (20 ± 1.0 g) were divided into a control group and an experimental group, with 15 fish in each group. The control group received an intraperitoneal injection of 30 μL of blank IgY antibody (30 μg), while the experimental group was immunized with 30 μL of VF17320 IgY antibody (30 μg). Meanwhile, the fish used for the *A. hydrophila* challenging experiment were treated the same. After 2 h, the challenge concentrations of *V. fluvialis* and *A. hydrophila* were 1 × 10^9^ CFU and 4.2 × 10^8^ CFU as preliminary LD_50_ determination experiment of fish bacterial challenge, respectively. The mortality of the fish was monitored for 14 consecutive days. The immune protection rate was calculated, and the immune activity was evaluated (25).

### 2.8 Active and active cross-protection rates of VF17320 DNA vaccine

The VF17320-pcDNA3.1 recombinant strain was expanded and cultured, followed by plasmid extraction and filtration using a 0.22 μm filter head to prepare the VF17320 DNA vaccine. The concentration of the fish immune plasmid was 1 μg/g, and the immune volume administered was 40 μL. For the *V. fluvialis* challenging experiment, the *C. auratus* (20 ± 1.0 g) were divided into a control group (blank pcDNA3.1 plasmid) and an experimental group (VF17320-pcDNA3.1 recombinant plasmid), with 15 fish in each group, and were immunized via intraperitoneal injection. Meanwhile, the fish used for the *A. hydrophila* challenging experiment were treated the same. Two immunizations were conducted on the 1st and 10th days, respectively. Seven days after the second immunization, the challenge concentrations of *V. fluvialis* and *A. hydrophila* were 8 × 10^8^ CFU and 4.0 × 10^8^ CFU as preliminary LD_50_ determination experiment of fish bacterial challenge, respectively. Observations and recordings were made over a period of 14 days. The immune protection rate was calculated, and the immune activity was evaluated (25).

### 2.9 Immune factors detection

Blood samples were collected from the tail vein of *C. auratus* 7 days after the second immunization with the VF17320 protein and its DNA vaccine, and from the subwing vein of laying hens 10 days after the third immunization with the VF17320 protein. The samples were then centrifuged to obtain serum. The levels of immune factors of acid phosphatase (ACP), alkaline phosphatase (AKP) and lysozyme (LZM) were evaluated according to the instructions provided by the detection kit (Sangon Biotechnology Co., Ltd., Shanghai, China) (26).

### 2.10 Kidney bacterial content

Two days after challenging the pathogenic bacteria, aseptically remove the kidney tissue from the *C. auratus* on an ultra-clean workbench, and the negative control was the kidney with no exposure to bacteria. Place the kidney in a homogenizer and homogenize it, then add 400 μL of physiological saline to prepare the homogenate. Next, take 200 μL of the mixed solution and spread it on LB solid medium. After incubating at 30°C for 1 h, invert the plates and continue the culture overnight. Finally, take photographs and count the number of colonies (27). The experiment was repeated three times.

### 2.11 Leukocyte phagocytosis analysis

Two days following the challenge of *C. auratus* with pathogenic bacteria, and ten days after laying hens were immunized for the third time with the VF17320 protein, plasma samples were collected from the tail vein of the *C. auratus* and the lower wing vein of the laying hens, respectively. Staphylococcus aureus was inactivated by adding 1% formaldehyde to physiological saline and heating at 80°C for 90 min. After inactivation, the cells were washed with physiological saline, centrifuged, and the supernatant was discarded. The remaining cells were resuspended in physiological saline to achieve an optical density (*OD*_600_) of 0.6. Subsequently, 0.2 mL of plasma from either the *C. auratus* or the chicken was mixed with 0.2 mL of the inactivated Staphylococcus aureus and incubated in a water bath at 25°C for 60 min. A 10 μL aliquot of the mixed solution was then used to prepare a blood smear, which was fixed with methanol. After the methanol evaporated, staining was performed using a fast Giemsa staining kit (Sangon Biotech Co., Ltd., Shanghai, China), and phagocytes were counted under a microscope. The phagocytosis percentage (*PP*%) was calculated using the formula: *PP*% = (number of cells participating in phagocytosis /100 phagocytes) × 100%. The phagocytosis index (*PI* %) was calculated as follows: *PI* % = (number of bacteria in phagocytes/number of cells participating in phagocytosis) × 100% (27).

### 2.12 Antioxidant factor analysis

Two days following the challenge with pathogenic bacteria, blood samples were collected from the tail vein of the *C. auratus* and subsequently centrifuged to isolate the serum. The levels of antioxidant factors, specifically superoxide dismutase (SOD), catalase (CAT), and malondialdehyde (MDA), were assessed in accordance with the instructions provided by the detection kit (Sangon Biotechnology Co., Ltd., Shanghai, China) (27).

### 2.13 mRNA expression levels of inflammatory factors

The real-time quantitative PCR (qRT-PCR) method was employed to assess the expression of inflammatory factor mRNA. On the second day post-challenge, kidney and spleen tissues from *C. auratus* were collected and placed in a mortar. Liquid nitrogen was added, and the tissues were ground using a pestle. RNA was then extracted following the instructions provided by the RNA extraction kit (Sangon Biotech Co., Ltd., Shanghai, China). Subsequently, cDNA was synthesized in accordance with the guidelines of the RNA extraction kit (Takara Biotechnology Co., Ltd., Beijing, China). The reaction protocol utilized a two-step method consisting of the following conditions: 95°C for 30 s, 95°C for 15 s, and 60°C for 30 s, repeated for 40 cycles. qRT-PCR was conducted using the SYBR^®^ Green Premix kit (Takar Biotechnology Co., Ltd., Beijing, China) along with synthetic primers (Supplementary Table 1), and the experiment was repeated three times (28).

### 2.14 Histopathological analysis

Two days after the *C. auratus* was challenged with pathogenic bacteria, the kidney, spleen, and small intestine tissues were fixed in Davidson’s fixative for over 18 h. Subsequently, the tissues were transferred to a 10% formaldehyde solution for more than 24 h and then subjected to a gradient ethanol solution (80, 90, 95, 100%) for 40 min, 20 min, 15 min, and 10 min, respectively. The tissues were then treated with xylene for transparency, using a 1:1 mixture of absolute ethanol and xylene, followed by xylene I and xylene II for 30 min each. Following this, the tissues were immersed in paraffin (a 1:1 mixture of paraffin and xylene) and treated with paraffin I, II, and III for 30 min each. After wax immersion, the tissues were placed into molds to solidify. They were then cut into 5 μm sections using a paraffin microtome and baked at 37°C for 24 h. The dried sections were stained with hematoxylin and eosin (H&E). The sections were deparaffinized in xylene I and II solutions for 10 min, rehydrated in a gradient of 100, 95, 80, and 70% ethanol for 5 min each, stained with hematoxylin for 5 min, rinsed with tap water, and differentiated in 1% hydrochloric acid alcohol for a few seconds. After rinsing with tap water, the sections were stained with eosin for 30 min, dehydrated in a gradient of ethanol (85, 95, 95%) for 10 s, and then dehydrated in absolute ethanol for 3 min. Following 5 min of transparency in xylene, the slides were sealed with neutral resin and observed under a microscope (28).

### 2.15 Immunofluorescence analysis of *Carassius auratus* kidney

The prepared kidney slices were deparaffinized in xylene three times. Following deparaffinization, rehydrate the slices in ethanol using a decreasing concentration gradient (100, 100, 95, 80, 50%). Wash the slices three times with phosphate-buffered saline (PBS). Next, soak the slices in 1% Triton X-100 for 30 min, followed by boiling in carbonate buffer (CBS) and allowing them to cool naturally. Subsequently, soak the slices in 3% hydrogen peroxide (H_2_O_2_) for 3 min and wash with PBS three times. Using an immunohistochemistry pen, draw a circle on the periphery of the tissue and then outline this circle. Add 50 μL (5%) of fetal bovine serum albumin (BSA) blocking solution and incubate at room temperature for 1.5 h. After washing with PBS, apply monoclonal antibodies against p53 or γH2A.X (1: 500) to the tissue and incubate overnight at 4°C. After another round of washing with PBS, introduce a 1: 500 diluted secondary antibody solution (Donkey anti-Rabbit IgG) and incubate at 37°C for 1 h. Finally, wash with PBS again, stain the nuclei with DAPI, and observe the slices under a fluorescence microscope (27).

### 2.16 Statistical analysis

All the experimental data were expressed as the mean ± SD. The significant difference from the respective control in all experiments was assessed by a one-way analysis of variance (ANOVA) using SPSS (IBM Corporation, Chicago, IL, United States). Values of *p* < 0.05 were considered statistically significant (28).

## 3 Results

### 3.1 Evaluation of active immune protection of VF17320 protein

#### 3.1.1 The expression, purification, and verify of VF17320 protein

To verify the expression and purification of the recombinant protein, VF17320 protein expressing strain were induced with IPTG, and a protein band with a molecular weight of 50.4 kDa was obtained, containing 30.0 kDa of VF17320 protein and 20.4 kDa fusion protein of pET-32a plasmid, which was consistent with the expected weight (Supplementary Figure 2A). The purified VF17320 protein was obtained using Ni-affinity chromatography (Supplementary Figure 2B).

Western blotting was performed to assess the accuracy of VF17320 expression and purification, and it showed a single band of VF17320 protein with the expected weight (Supplementary Figure 2C), indicating that VF17320 protein was purification successfully.

#### 3.1.2 Active and active cross-protection rates of VF17320 protein

**Figure 1:**
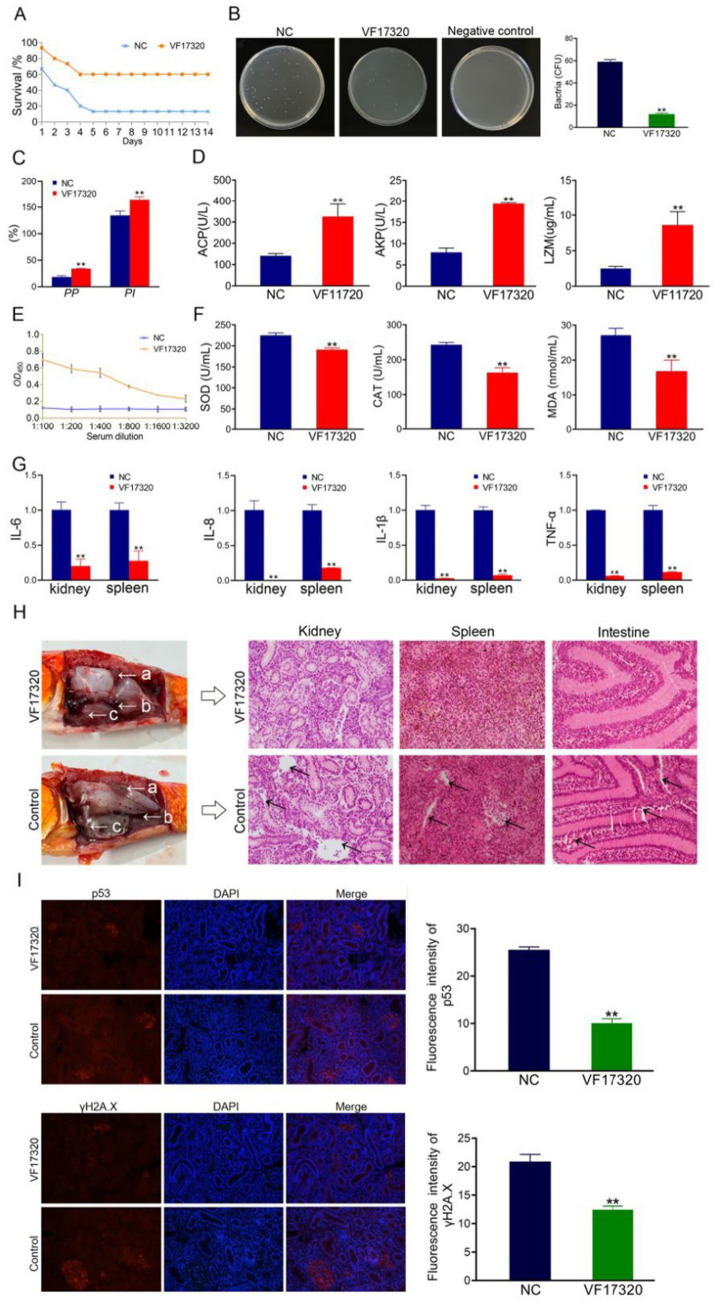
Active immune protection from VF17320 protein immunization in *C. auratus*. **(A)** Survival rate with active immune protection against *V. fluvialis.* **(B)** Kidney bacteria count after challenge. **(C)** Plasma leukocyte phagocytosis activity in *C. auratus*. **(D)** *C. auratus* serum immune indexes. **(E)** Mutual recognition between *C. auratus* serum and *V. fluvialis in vitro*. **(F)** The expression levels of antioxidation-related factors in *C. auratus* serum. **(G)** Inflammatory factor mRNA expression levels. **(H)** Histopathological kidney, spleen, and intestine sections of *C. auratus*; (a) kidney, (b) spleen, and (c) intestine. **(I)** Immunofluorescence analysis of renal p53 and γH2A.X in *C. auratus*. Compared with control group, *p < 0.05 and **p < 0.01.

**Figure 2:**
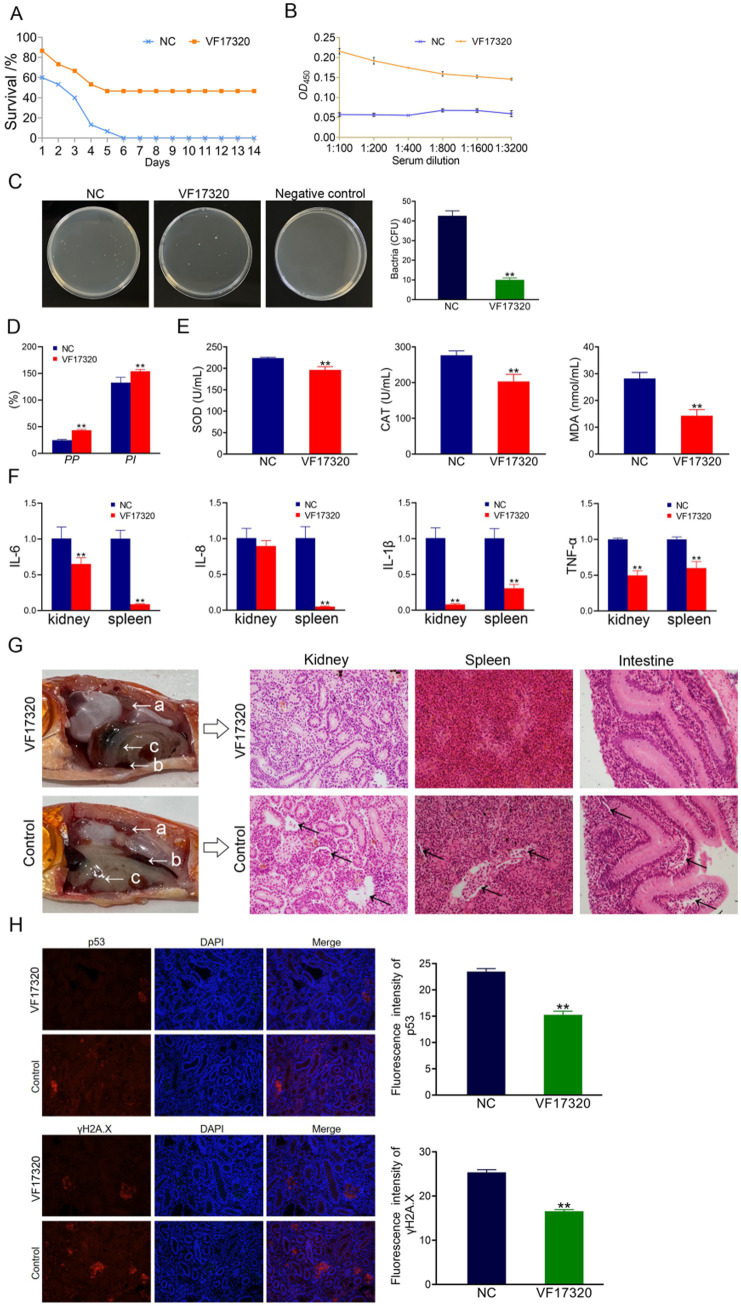
Active cross-protection from VF17320 protein immunization in *C. auratus*. **(A)** Survival rate with active immune protection against *A. hydrophila*. **(B)** *In vitro* recognition of *A. hydrophila* and *C. auratus* serum. **(C)** Kidney bacteria count after challenge. **(D)** Plasma leukocyte phagocytosis activity in *C. auratus*. **(E)** The expression of antioxidant-related factors in *C. auratus* serum. **(F)** Inflammation-related mRNA expression. **(G)** Histopathological kidney, spleen, and intestine sections of *C. auratus*; (a) kidneys, (b) spleen, and (c) small intestine. **(H)** p53 and γH2A.X expression levels in *C. auratus* kidney after challenge. Compared with control group, *p < 0.05 and **p < 0.01.

After immunization of *C. auratus* with VF17320 protein, the challenge results indicated that following exposure to *V. fluvialis* and *A. hydrophila*, the *C. auratus* exhibited reduced swimming activity, epidermal bleeding, abdominal swelling, and a significant mortality rate. The mortality rate stabilized after six days (Figures 1A, 2A). The active and active cross-immune protection rates of VF17320 against *V. fluvialis* and *A. hydrophila* were 70% (*p* < 0.01) and 53.33% (*p* < 0.01), respectively. This suggests that VF17320 provides effective active and active cross-immune protection.

#### 3.1.3 Bacterial count in *Carassius auratus* kidney of VF17320 protein

The results of the bacterial counting experiment indicated that the number of kidney bacteria in the *C. auratus* immune group immunization with VF17320 protein was significantly reduced after exposure to *V. fluvialis* and *A. hydrophila* (*p* < 0.01) (Figures 1B, 2C) compared to the control group. These findings suggest that VF17320 protein confers immunity that effectively inhibits bacterial infection in kidney tissue.

#### 3.1.4 Leukocyte phagocytosis in *Carassius auratus* plasma of VF17320 protein

The leukocyte phagocytosis experiment indicated that both the leukocyte phagocytosis index (*PI*) and the phagocytosis percentage (*PP*) in the plasma of *C. auratus* from the VF17320 immunized group exhibited significant increases (*p* < 0.01) (Figures 1C, 2D). This suggests that VF17320 immunity enhances the phagocytic activity of leukocytes in *C. auratus* plasma.

#### 3.1.5 Immune factors in *Carassius auratus* serum of VF17320 protein

The detection results for immune factors revealed that the levels of ACP, AKP, and LZM in the serum of *C. auratus* from the VF17320 immunized group were significantly elevated (*p* < 0.01) (Figure 1D). This indicates that the outer membrane protein VF17320 can activate non-specific immunity in *C. auratus*.

#### 3.1.6 *In vitro* interaction detection of VF17320 protein

The ELISA results indicated that the serum from *C. auratus* in the VF17320 immunization group could bind to *V. fluvialis* and *A. hydrophila*, with absorbance decreasing as antibody dilution increases (Figures 1E, 2B). These results suggest that *C. auratus* serum interacts with *V. fluvialis* and *A. hydrophila in vitro*.

#### 3.1.7 Antioxidant-related factors (SOD, CAT, and MDA) in *Carassius auratus* serum

The results of the antioxidant factor detection revealed that the VF17320 immune group exhibited a significant reduction in most antioxidant-related factors (SOD, CAT, and MDA) in the *C. auratus* serum after challenging with *V. fluvialis* and *A. hydrophila* (*p* < 0.01) (Figures 1F, 2E) compared to the control group. These findings indicate that VF17320 can resist infection in *C. auratus* caused by *V. fluvialis* and *A. hydrophila*.

#### 3.1.8 mRNA expression related to inflammation in *Carassius auratus* of VF17320 protein

The mRNA expressions of IL-6, IL-8, TNF-*α*, and IL-1β in the kidneys and spleens of the VF17320 immunized group were significantly reduced (*p* < 0.01) (Figures 1G, 2F) compared to the control group. These results indicate that VF17320 can mitigate the inflammatory response in *C. auratus* induced by *V. fluvialis* and *A. hydrophila*.

#### 3.1.9 Histopathological morphological observation in *Carassius auratus* of VF17320 protein

The histopathological sections indicated that in the control group, the kidney tissue structure was loose and incomplete, exhibiting parenchymal damage, severe cellular vacuolization, and apoptosis. Similarly, the spleen tissue was also found to be incomplete, with a reduced cell density and evidence of apoptosis. Furthermore, the intestinal mucosal lamina propria displayed atrophy, and the villous structure had collapsed. The VF17320 immunized group demonstrated intact and well-defined structures in the kidneys, spleens, and intestines (Figures 1H, 2G). These findings suggest that VF17320 immunization can preserve the integrity of the internal organs in *C. auratus*.

#### 3.1.10 Immunofluorescence analysis of *Carassius auratus* kidney of VF17320 protein

The immunofluorescence analysis revealed that p53 and γH2A.X were labeled in red fluorescence, while the DAPI-stained nuclei appeared blue fluorescence. Compared to the control group, the expression levels of p53 and γH2A.X were notably decreased (*p* < 0.01) (Figures 1I, 2H). The results indicate that active immunization with VF17320 can effectively reduce apoptosis and DNA damage in the kidney cells of *C. auratus*.

### 3.2 Evaluation of IgY antibody passive immunity abilities

#### 3.2.1 Leukocyte phagocytosis in chicken plasma of VF17320 IgY antibody

**Figure 3:**
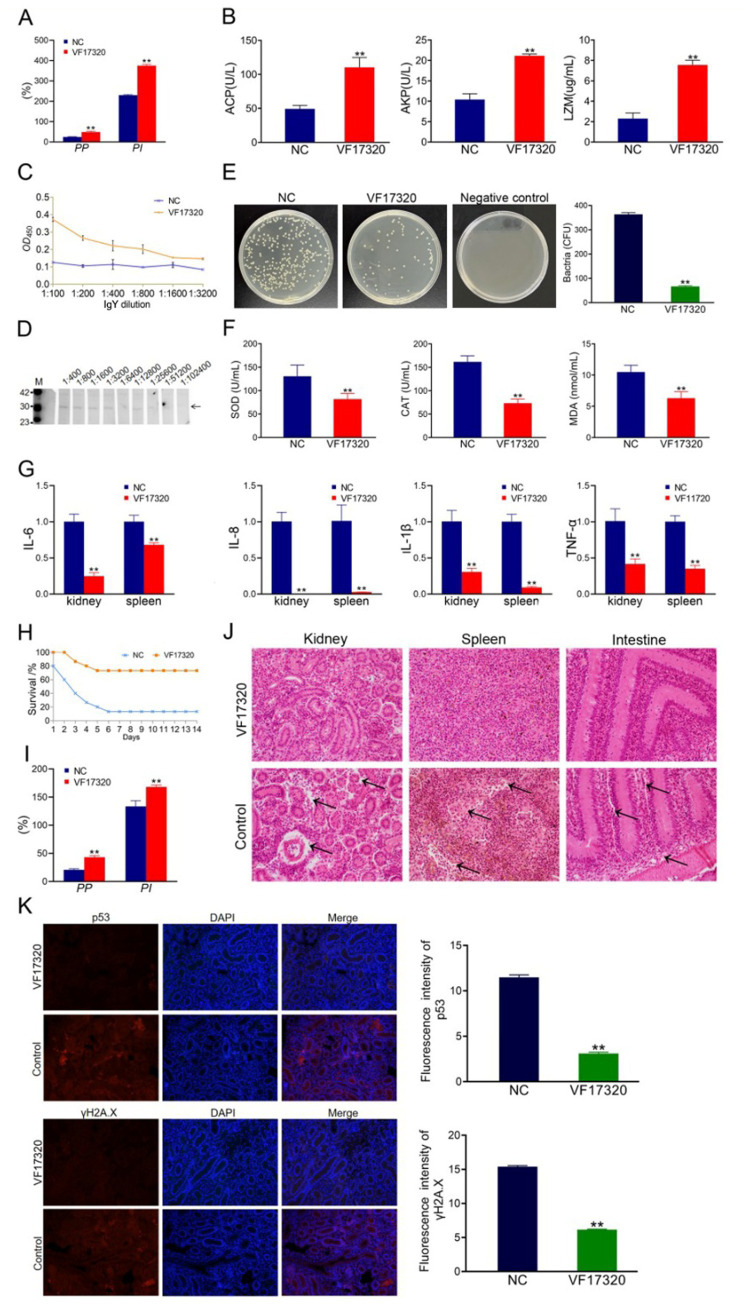
Passive protection due to VF17320 IgY immunization in *C. auratus*. **(A)** Phagocytosis activity of chicken plasma leukocyte. **(B)** Chicken serum immune index. **(C)** Mutual recognition of IgY and *V. fluvialis* in vitro. **(D)** IgY titer. **(E)** *C. auratus* kidney bacteria count after *V. fluvialis* challenge. **(F)** Differences in antioxidation-related factor expression levels in the *C. auratus* serum. **(G)** mRNA expression of inflammatory factors. **(H)** Survival rate with passive immune protection against *V. fluvialis*. **(I)** *C. auratus* plasma leukocyte phagocytosis activity. **(J)** *C. auratus* kidney, spleen, and intestine histopathological sections. **(K)** Immunofluorescence analyses for p53 and γH2A.X in *C. auratus* renal. Compared with control group, *p < 0.05 and **p < 0.01.

The cell phagocytosis experiment indicated that both the phagocytosis index and the percentage of phagocytosis among chicken plasma leukocytes in the VF17320 immunized group were significantly increased (*p* < 0.01) (Figure 3A). These results suggest that the VF17320 effectively activates the phagocytosis of chicken plasma leukocytes.

#### 3.2.2 Immune factors in chicken serum of VF17320 IgY antibody

The immune factors of ACP, AKP, and LZM in the serum of chickens from the VF17320 immunized group were significantly elevated (*p* < 0.01) (Figure 3B). Thus, the VF17320 can stimulate an immune response in chickens.

#### 3.2.3 *In vitro* interaction analysis of VF17320 IgY antibody

To simulate the *in vitro* interaction between the VF17320 IgY antibody and bacteria, ELISA experiments were conducted. The results demonstrated that the VF17320 IgY antibody could bind to *V. fluvialis*, with absorbance decreasing as the antibody dilution increased (Figure 3C). Thus, the VF17320 IgY antibody can interact with *V. fluvialis in vitro*.

#### 3.2.4 Antibody titers and specificity of VF17320 IgY antibody

To assess the potency and specificity of the VF17320 IgY antibody, Western blotting was conducted using gradient dilutions of the antibody. The results indicated the presence of a band, confirming the specificity of the VF17320 IgY antibody, with a titer reaching 1: 25600 (Figure 3D).

#### 3.2.5 Passive and passive cross-protection rates to *Carassius auratus* of VF17320 IgY antibody

**Figure 4:**
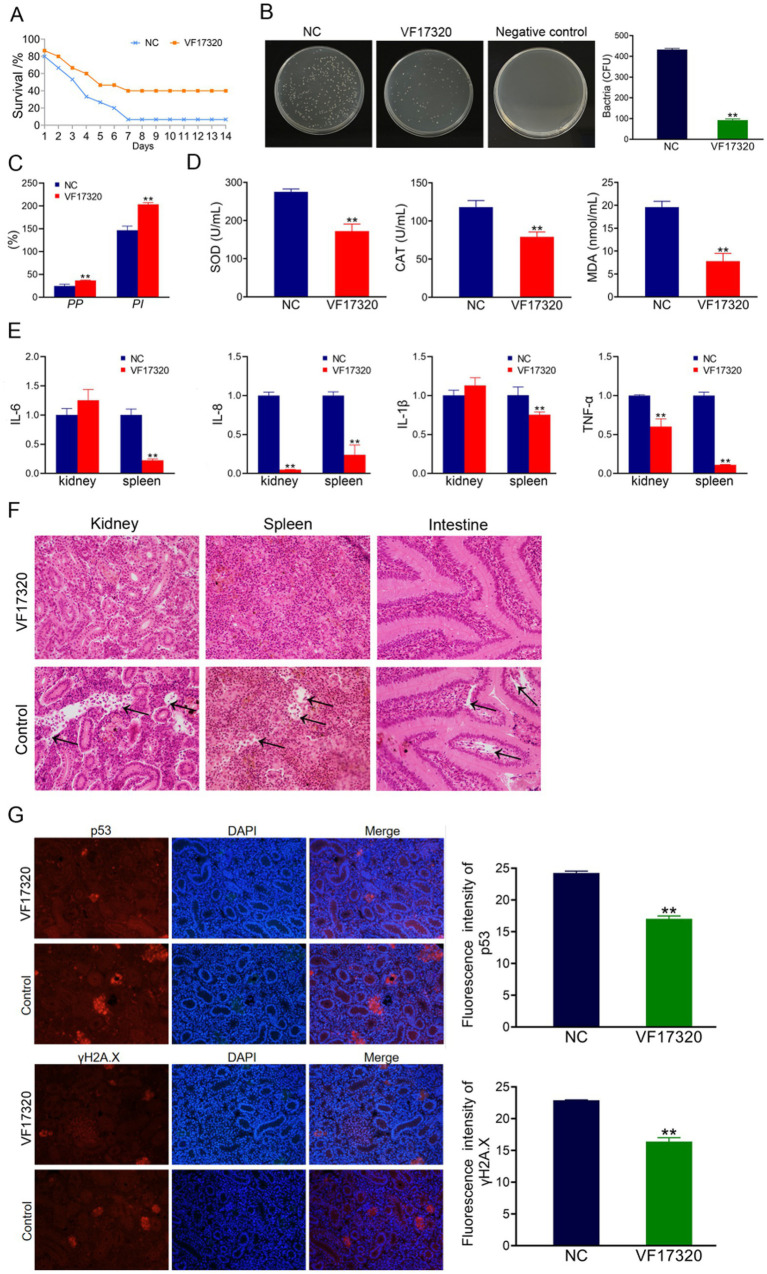
Passive cross-protection due to VF17320 IgY immunization in *C. auratus*. **(A)** *C. auratus* survival rate with immune protection against *A. hydrophila*. **(B)** Kidney bacteria count after the challenge. **(C)** *C. auratus* plasma leukocyte phagocytosis activity. **(D)** Differences in the of antioxidant-related factor expression levels in the *C. auratus* serum. **(E)** Inflammation-related mRNA expression. **(F)** *C. auratus* kidney, spleen and intestine histopathological sections. **(G)** p53 and γH2A.X expression levels in *C. auratus* kidney after challenge. Compared with control group, *p < 0.05 and **p < 0.01.

After passive immunization with VF17320 IgY antibody and challenging to bacteria, *C. auratus* exhibited symptoms such as slow swimming, epidermal bleeding, abdominal swelling, and significant mortality. The mortality rate stabilized after six days (Figures 3H, 4A). Furthermore, the passive and passive cross-immune protection rates of the VF17320 IgY antibody against *V. fluvialis* and *A. hydrophila* were 73.33% (*p* < 0.01) and 60% (*p* < 0.01), respectively. Thus, the VF17320 IgY antibody possesses passive and passive cross-protective effects.

#### 3.2.6 Kidney bacteria in *Carassius auratus* of VF17320 IgY antibody

The bacterial counting test revealed that the number of bacteria in *C. auratus* kidneys from the VF17320 IgY immunized group was significantly reduced after challenging to *V. fluvialis* and *A. hydrophila* (*p* < 0.01) compared to the control group (Figures 3E, 4B). These findings suggest that the VF17320 IgY antibody effectively inhibits bacterial infection in kidney tissue.

#### 3.2.7 Plasma leukocyte phagocytosis in *Carassius auratus* of VF17320 IgY antibody

The leukocyte phagocytosis test indicated that the leukocyte phagocytosis index (*PP*%) and the phagocytosis percentage (*PI*%) in the VF17320 IgY immunized group significantly increased (*p* < 0.01) compared to the control group (Figures 3I, 4C). The results suggest that the VF17320 IgY antibody enhance the phagocytosis activity of plasma leukocytes in *C. auratus*.

#### 3.2.8 Antioxidant-related factors in *Carassius auratus* serum of VF17320 IgY antibody

The results of the antioxidant factor analysis revealed that, following challenges with *V. fluvialis* and *A. hydrophila*, the VF17320 IgY immune group exhibited a significant reduction in most antioxidant-related factors (SOD, CAT, and MDA) compared to the control group (*p* < 0.01) (Figures 3F, 4D). These findings indicate that the VF17320 IgY antibody can enhance antioxidation of *C. auratus* induced by *V. fluvialis* and *A. hydrophila*.

#### 3.2.9 mRNA expression related to inflammation in *Carassius auratus* of VF17320 IgY antibody

The mRNA expressions related to inflammation-related gene of IL-6, IL-8, TNF-*α*, and IL-1β in the kidneys and spleens in VF17320 IgY immunized group were significantly reduced (*p* < 0.01) compared to the control group (Figures 3G, 4E). These results suggest that the VF17320 IgY antibody can diminish the inflammatory response in *C. auratus* induced by *V. fluvialis* and *A. hydrophila*.

#### 3.2.10 Histopathological morphological observation in *Carassius auratus* of VF17320 IgY antibody

In the control group, the histopathological section indicated that the kidney tissue exhibited a loose and incomplete structure with atrophied and degenerated glomeruli and renal tubules, alongside evidence of cellular apoptosis. Similarly, the spleen tissue appeared incomplete, characterized by reduced cell density and the occurrence of apoptosis. Furthermore, the intestinal mucosal lamina propria demonstrated atrophy and an incomplete structure, along with signs of apoptosis. In contrast, the VF17320 IgY immunized group displayed intact and well-defined structures in the kidney, spleen, and intestine (Figures 3J, 4F). These findings suggest that VF17320 IgY immunity can effectively preserve the structural integrity of the visceral tissues in *C. auratus*.

#### 3.2.11 Kidney immunofluorescence analysis of *Carassius auratus* of VF17320 IgY antibody

The immunofluorescence results revealed that the expression levels of p53 and γH2A.X in VF17320 IgY immunized group were decreased (*p* < 0.01) compared to the control group (Figures 3K, 4G). The results indicated that passive immunization with the VF17320 IgY antibody could mitigate apoptosis and DNA damage in the kidney cells of *C. auratus*.

### 3.3 Evaluation of DNA vaccine active immune protection

#### 3.3.1 Active and active cross-protection rates of *Carassius auratus* of VF17320 DNA vaccine

**Figure 5:**
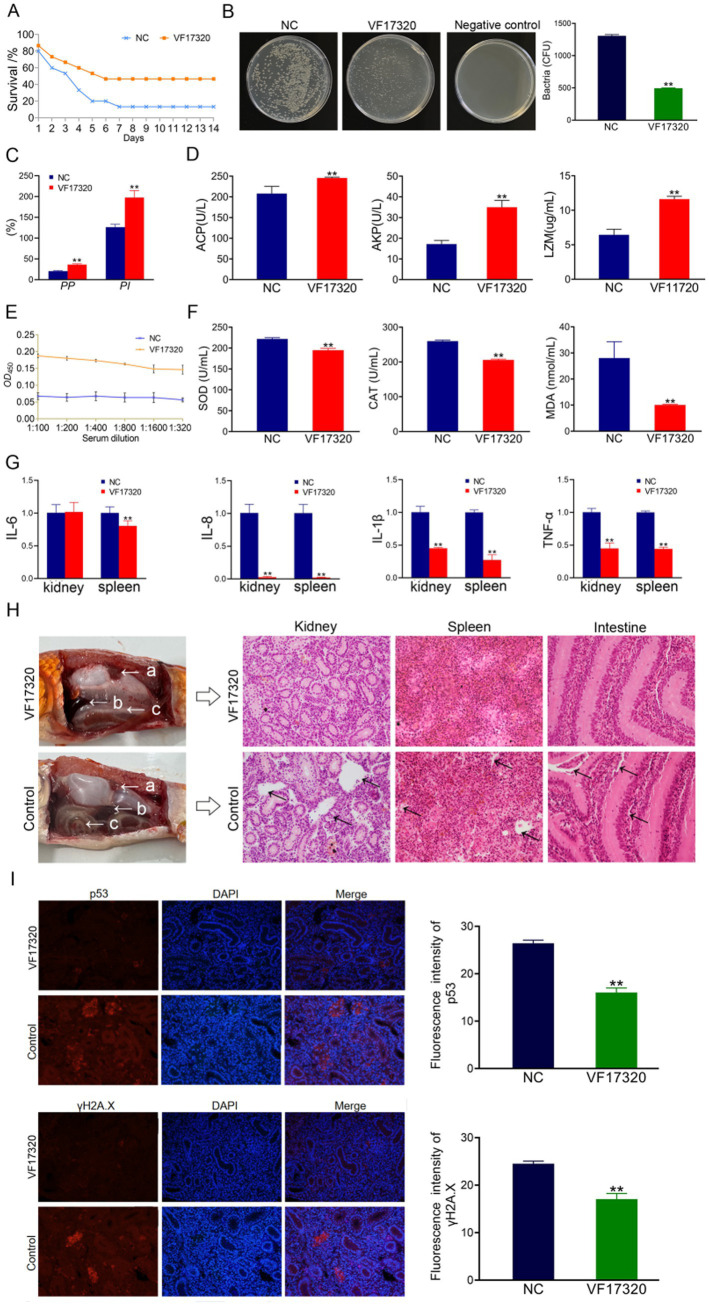
Active immune protection due to VF17320 DNA vaccine in *C. auratus*. **(A)** Survival rate with active immune protection against *V. fluvialis*. **(B)** Kidney bacteria count after challenge. **(C)** Plasma leukocyte phagocytosis activity in *C. auratus*. **(D)** Serum immune index detection in *C. auratus*. **(E)** The recognition of *V. fluvialis* and *C. auratus* serum. **(F)** Antioxidation-related factors expression differences in *C. auratus* serum. **(G)** Inflammation-related mRNA expression. **(H)** Histopathological kidney, spleen, and intestine sections of *C. auratus*: (a) kidneys, (b) spleen, and (c) small intestine. **(I)** p53 and γH2A.X expression levels in *C. auratus* kidney after challenge. Compared with control group, *p < 0.05 and **p < 0.01.

**Figure 6:**
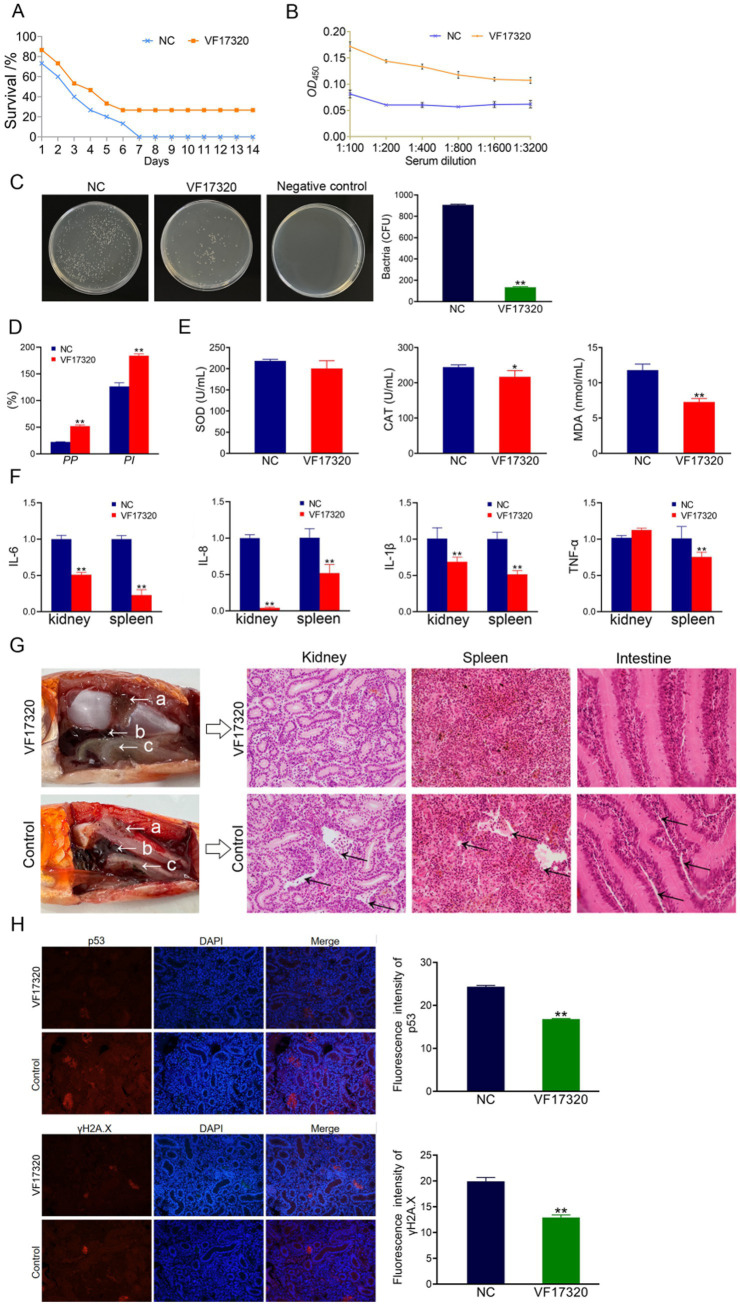
Active cross-protection due to VF17320 DNA vaccine in *C. auratus*. **(A)** Survival rate with active immune protection against *A. hydrophila*. **(B)** The recognition of *A. hydrophila* and *C. auratus* serum. **(C)** Kidney bacteria count after challenge. **(D)** Plasma leukocyte phagocytosis activity in *C. auratus*. **(E)** Antioxidant-related factor expression differences in *C. auratus* serum. **(F)** Inflammation-related mRNA expression. **(G)** Histopathological kidney, spleen, and intestine sections of *C. auratus*: (a) kidney, (b) spleen, and (c) small intestine. **(H)** p53 and γH2A.X expression levels in *C. auratus* kidney after challenge. Compared with control group, *p < 0.05 and **p < 0.01.

The results of the challenge test demonstrated that following the VF17320 DNA vaccine immunization against *V. fluvialis* and *A. hydrophila*, *C. auratus* exhibited slow swimming movements, epidermal hemorrhage, and abdominal swelling. The mortality rate stabilized after six days (Figures 5A, 6A). Furthermore, the immune protection rates of the VF17320 DNA vaccine against *V. fluvialis* and *A. hydrophila* were 80% (*p* < 0.01) and 66.67% (*p* < 0.01), respectively. These findings indicate that the VF17320 DNA vaccine possesses active and active cross-protective effects.

#### 3.3.2 The number of bacteria in *Carassius auratus* kidney of VF17320 DNA vaccine

The bacterial culture test revealed that the bacterial concentration in the kidneys of *C. auratus* from the VF17320 DNA vaccine immunized group was significantly reduced (*p* < 0.01) compared to the control group (Figures 5B, 6C). Thus, the VF17320 DNA vaccine can mitigate kidney infections caused by *V. fluvialis* and *A. hydrophila* in *C. auratus*.

#### 3.3.3 Leukocyte phagocytosis in *Carassius auratus* plasma of VF17320 DNA vaccine

The leukocyte phagocytosis experiment indicated that the phagocytic index and the percentage of phagocytosis of *C. auratus* plasma in VF17320 DNA vaccine immunized group were significantly elevated (*p* < 0.01) compared to the control group (Figures 5C, 6D). Thus, the VF17320 DNA vaccine effectively activates leukocyte phagocytosis in *C. auratus*.

#### 3.3.4 Immune factors in *Carassius auratus* serum of VF17320 DNA vaccine

The immune factors of ACP, AKP, and LZM in *C. auratus* serum from the VF17320 DNA vaccine immunized group were significantly elevated (*p* < 0.01) (Figure 5D). These findings suggest that the VF17320 DNA vaccine is effective in activating the immune response in *C. auratus*.

#### 3.3.5 *In vitro* interaction analysis of VF17320 DNA vaccine

The ELISA results demonstrated that the *C. auratus* serum from the VF17320 immunized group exhibited an interaction ability to *V. fluvialis* and *A. hydrophila*, and the absorbance decreased with increasing antibody dilution (Figures 5E, 6B). These results suggest that *C. auratus* serum can interact with *V. fluvialis* and *A. hydrophila in vitro*.

#### 3.3.6 Antioxidant-related factors in *Carassius auratus* serum of VF17320 DNA vaccine

The antioxidant factors (SOD, CAT, and MDA) exhibited a significant reduction in the VF17320 immune group following challenge with *V. fluvialis* and *A. hydrophila* (*p* < 0.01) compared to the control group (Figures 5F, 6E). The results imply that the VF17320 DNA vaccine can confer a resistance caused by *V. fluvialis* and *A. hydrophila* in *C. auratus*.

#### 3.3.7 mRNA expression related to inflammation in *Carassius auratus* of VF17320 DNA vaccine

The mRNA expressions of inflammation-related genes (IL-6, IL-8, TNF-*α*, and IL-1β) in the kidneys and spleens of the VF17320 immunized group were significantly reduced (*p* < 0.01) compared to the control group (Figures 5G, 6F). These findings suggest that the VF17320 DNA vaccine effectively mitigates the inflammatory response in *C. auratus* induced by *V. fluvialis* and *A. hydrophila*.

#### 3.3.8 Histopathological morphological observation of *Carassius auratus* of VF17320 DNA vaccine

In the control group, histopathological observations of *C. auratus* revealed that the kidney tissue exhibited a loose and incomplete structure, with atrophic and degenerated glomeruli and renal tubules, alongside apoptotic cells; similarly, the spleen tissue appeared incomplete with reduced cell density and evidence of apoptosis; additionally, the intestinal mucosal lamina propria was atrophied and displayed incomplete structure along with apoptosis. In contrast, the structures of the kidney, spleen, and intestine in the VF17320 immunized group were intact and well-defined (Figures 5H, 6G). This indicates that the VF17320 DNA vaccine helps maintain the integrity of the internal structures of *C. auratus*.

#### 3.3.9 Kidney immunofluorescence analysis in *Carassius auratus* of VF17320 DNA vaccine

Immunofluorescence analysis of *C. auratus* kidneys demonstrated that the expression levels of p53 and γH2A.X in the VF17320 immunized group were decreased (*p* < 0.01) compared to the control group (Figures 5I, 6H). The results indicate that active immunization with the VF17320 DNA vaccine can reduce apoptosis and DNA damage in the kidney cells of *C. auratus*.

## 4 Discussion

Vaccination is recognized as an effective intervention against bacterial and viral diseases. Vaccines play a crucial role to control pathogens in aquaculture (29, 30). The primary types of vaccines currently employed in aquaculture include attenuated vaccines, inactivated vaccines, protein subunit vaccines, DNA vaccines, and mRNA vaccines (31). Bacterial outer membrane proteins (OMPs) exhibit significant immunogenicity and can be utilized to develop multivalent vaccines (32), and has garnered considerable attention in the development of protein vaccines. Therefore, identifying OMPs with effective passive immune protection capabilities is particularly important (33). In this study, the protein vaccine, DNA vaccine, and egg yolk antibody (IgY) vaccine derived from the *V. fluvialis* outer membrane protein VF17320 were used to immunize *C. auratus*. The immune response was evaluated through various methods, including immune activity analysis, protection rate tests, assessments of anti-inflammatory and antioxidant effects, histopathology, and immunofluorescence, to determine the vaccines’ immunological efficacy against *V. fluvialis* and *A. hydrophila*.

The immune protection rate serves as an intuitive metric for evaluating immune protection capabilities. Duan et al. immunized *Anguilla japonica* with the *Edwardsiella* outer membrane protein A (OmpA) vaccine and subsequently challenged the fish with the virus, reporting a relative survival rate (RPS) of 77.7% (*p* < 0.05) for the OmpA group (34). Kunza et al. prepared recombinant protein vaccines, SpaO and LamB, using aluminum hydroxide gel precipitation, and immunized mice with these formulations. The results indicated that the combination of rSpaO and rLamB provided 80% protection, whereas the monovalent rSpaO and rLamB vaccines offered protection rates of 60 and 40%, respectively. These findings suggest that the combined rSpaO and rLamB vaccine effectively enhances the immune response compared to monovalent vaccines, offering superior protection against pathogen infection (35). Additionally, Kim et al. developed a multi-pathogen DNA vaccine utilizing a dual expression system based on the *PA-D4* gene from *Bacillus anthracis* and the *hct* gene from *Clostridium botulinum*, and confirmed that the vaccine provided over 50% protective rate. This work contributes to the development of strategies for potential vaccines against biothreat agents (36). Furthermore, Liang et al. prepared a lysate vaccine from an *A. hydrophila* TPS strain, immunizing crucian carp and subsequently challenging them. The high-concentration of bacteria lysate vaccine group (1 × 10^8^ CFU/mL) exhibited an immune protection rate of 88.89%. Overall, the development of the TPS phage lysate vaccine significantly enhanced the immunity of crucian carp, providing a higher level of protection and establishing a foundation for future phage aquatic vaccine development (37). This study developed active immunity vaccines, including DNA vaccines and IgY antibody vaccines, targeting the outer membrane protein VF17320 of *V. fluvialis*. *C. auratus* were utilized for the evaluation of immune protection. The results demonstrated that the immune protection rate of the VF17320 vaccine against *V. fluvialis* was 60% (*p* < 0.01), while the protection rate against *A. hydrophila* was 53.33% (*p* < 0.01). Additionally, the IgY vaccine of VF17320 exhibited immune protection rates of 73.33% (*p* < 0.01) against *V. fluvialis* and 60% (*p* < 0.01) against *A. hydrophila*. Furthermore, the immune protection rates of the VF17320 DNA vaccine were 80% (*p* < 0.01) against *V. fluvialis* and 66.67% (*p* < 0.01) against *A. hydrophila*. In this research, the mortality of fish was checked in 14 days, and lacked the assessment of the durability of the immune response. Long-term efficacy, memory response, or booster potential are important to the vaccine, and did not explore in this study. Although according to the survival rate testing criteria, it is only necessary to observe the mortality of fish for 14 days (27, 28). For the development of vaccines, it is necessary to carry out the assessment of long-term immune memory in future study. These findings indicate that three vaccines of VF17320 provide significant immune protection and demonstrate cross-immune activity.

Bacterial coating allows for the visual observation of bacterial infections in animals. Han et al. prepared IgY antibodies against ectotoxin-producing *Escherichia coli* (ETEC) and immunized ETEC-infected mice with these antibodies. By analyzing fecal samples, it was observed that the bacterial count in the ETEC IgY-immunized group was significantly reduced (38). Miller et al. utilized knowledge of the biology and virulence of *M. gallisepticum* MG to develop a subunit vaccine comprising GapA, CrmA, and VlhAs. This vaccine was administered to chickens, which were subsequently challenged with the Rlow virus strain. The bacterial coating test revealed a significant reduction in bacterial numbers in the tracheal samples of the immunized group (39). Pen et al. immunized mice with the prokaryotic expression of the outer membrane protein rOmpA of *E. coli* and subsequently challenged them with the virus. Their bacterial coating test demonstrated a significant reduction in the bacterial count in the fecal samples of the immunized group (40). In this study, we immunized *C. auratus* with the VF17320 protein vaccine, DNA vaccine, and IgY antibody vaccine, followed by challenge tests with *V. fluvialis* and *A. hydrophila*. Through kidney tissue coating, we found that the bacterial count in the kidney tissue of the VF17320-immunized group was significantly reduced (*p* < 0.01). These results indicate that the three VF17320 vaccines can effectively mitigate kidney infections in *C. auratus* caused by *V. fluvialis* and *A. hydrophila*.

Leukocyte phagocytosis and immune factors are commonly employed to evaluate non-specific indicators in the serum (41, 42). Kordon et al. prepared an attenuated vaccine (*Edwardsiella ictaluri*) and administered it to *Channel Catfish*, finding that the vaccine enhanced active phagocyte uptake, improved cell phagocytosis activity, induced bactericidal activity, and promoted both early and late apoptosis in catfish B cells. These findings indicate that the vaccine activates the innate immunity of catfish (43). Liu et al. utilized the *Nocardia seriolae* strain ΔNsAld as a live vaccine to immunize hybrid snakehead fish, detecting significant increases in the levels of AKP and ACP, which suggests that the vaccine can induce both humoral and cell-mediated immune responses. The results indicate that strain ΔNsAld may serve as a potential candidate for the development of live vaccines aimed at controlling fish nocardiosis in aquaculture (44). Zhang et al. immunized *Misgurnus anguillicaudatus* with the *Aeromonas veronii* live attenuated vaccine ΔhisJ, leading to increased enzyme activity parameters (SOD, LZM, ACP, and AKP) in skin mucus and serum, as well as elevated levels of specific IgM antibodies and cytokine IL-1β. These findings demonstrate that the live attenuated vaccine is suitable for the development of a safe and effective vaccine against *A. veronii* infection in *M. anguillicaudatus* within aquaculture (45). Zhao et al. prepared the OMP Aha1 *Lactobacillus casei*-pPG1-Aha1 recombinant (Lc-pPG1-Aha1) from *A. hydrophila* and subsequently immunized carp with it. The study found that the phagocytic activity of carp cells was enhanced, with significant increases in serum antibodies, AKP, ACP, SOD, LZM, and complement components C3 and C4 levels. Moreover, immune-related gene expression was markedly upregulated in the liver, spleen, and intestine, indicating that Lc-pPG1-Aha1 activates the innate immunity of carp. Recombinant *Lactobacillus casei* strains may serve as promising candidates for vaccines against *A. hydrophila* infection in carp (46). In this study, after *C. auratus* were immunized with the VF17320 protein vaccine, DNA vaccine, and IgY antibody vaccine, significant increases in immune factor indicators such as LZM, ACP, and AKP in the serum were observed (*p* < 0.01). Additionally, plasma levels of PI and PP also increased significantly (*p* < 0.01). These findings suggest that the three vaccines enhanced the phagocytic activity of *C. auratus* and chicken plasma cells. In summary, these three vaccines effectively activated non-specific immunity in both *C. auratus* and chickens.

ELISA and Western blotting are widely utilized techniques for detecting the specific binding of antigens and antibodies *in vitro* (47, 48). Western blotting analysis demonstrated that the transcription and protein levels of the host factor FoxJ1 were significantly down-regulated in primary porcine alveolar macrophages (PAM) infected with ASFV (49). Furthermore, the analysis indicated that FoxJ1 facilitates the degradation of ASFV through the autophagy pathway. The findings regarding MGF505-2R and E165R proteins contribute to the development of antiviral drugs or vaccines targeting ASFV infection. Sapp et al. conducted a study on the detection and evaluation of rodent host antibody responses to Baylisascaris-specific antigens using Western Blotting and ELISA. The specificity of Western Blotting was found to be 100%, while that of ELISA was 94.1% (50). Felegary et al. prepared recombinant chimeric protein IgY antibodies targeting *Shigellosis* antigens IpaD, IpaB, StxB, and VirG, and assessed the preventive efficacy of IgY by immunizing mice and subsequently challenging them with the virus. The specificity of the IgY antibodies was confirmed through Western Blotting. ELISA results indicated that the IgY antibodies could recognize and react with the recombinant proteins, achieving a detected antibody titer of 1:25600 (51). This study also employed ELISA to investigate the in vitro interaction between bacteria and IgY antibodies/*C. auratus* serum, revealing a significant interaction. Furthermore, using the whole bacterial protein of *V. fluvialis* as the antigen, the specificity of the IgY antibody was tested via Western Blotting. It was determined that the IgY antibody binds to the corresponding site of the protective protein of *V. fluvialis*, with the titer reaching 1:25600. These findings suggest that the immunity conferred by the three VF17320 vaccines, activates specific immunity in both laying hens and *C. auratus*.

Anti-inflammatory and antioxidant activities play a crucial role in helping the body combat bacterial infections (52, 53). The effects of these activities can be assessed through the expression of inflammatory and antioxidant factors (54). Zhang et al. (55) administered anti-*Streptococcus agalactiae*-specific IgY antibodies to tilapia over a period of 10 days, followed by a challenge with *S. agalactiae*. The analysis of serum revealed a reduction in malondialdehyde (MDA) levels within the immune group, indicating enhanced antioxidant activity. Furthermore, the expression levels of inflammatory factors demonstrated that specific IgY can down-regulate IL-8 and TNF-*α* gene expression, thereby mitigating the inflammatory response. Collectively, these findings suggest that specific IgY warrants further investigation as a potential alternative to antibiotics for treating infections caused by gastrointestinal pathogens. Additionally, *Lates calcarifer* was immunized with monovalent and bivalent vaccines targeting *Streptococcus iniae* and *Vibrio harveyi*, resulting in significantly reduced serum MDA levels in the immunized group. The bivalent vaccines were effective in decreasing oxidative stress in Asian seabass (56). Vieira et al. investigated the antioxidant levels in the saliva of elderly individuals vaccinated against COVID-19, revealing a significant reduction in total peroxide levels within the ChadoX-1 group. Additionally, the levels of TNF-α, IL-6, IL-12p70, and the IL-12p70/IL-10 ratio were also decreased, suggesting that the ChadoX-1 group had a notable impact on the immune and inflammatory response as well as redox balance (57). In this study, we immunized *C. auratus* with the VF17320 protein vaccine, DNA vaccine, and IgY antibody vaccine, subsequently conducting challenge tests with *V. fluvialis* and *A. hydrophila*. By measuring serum antioxidant indicators and levels of inflammatory factors, we found that most antioxidant indicators exhibited significant decreases (*p* < 0.05), while most inflammatory factor mRNA indicators (IL-1β, TNF-α, IL-6, IL-8) showed extremely significant reductions (*p* < 0.01). These findings indicate that oxidative damage and inflammatory responses to pathogenic bacteria in *C. auratus* were diminished. Thus, it can be concluded that the three VF17320 vaccines, possess anti-inflammatory and antioxidant properties.

The structure and function of animal tissues are crucial for their resistance to bacterial infections (58). Histopathology serves as a visual representation of damage to animal tissues and can be utilized to assess the immune capacity of new drugs (59, 60). Zels et al. analyzed the histopathological lesions of the placenta following vaccination with the COVID-19 vaccine through histopathological sections. Their findings indicated that the placentas of unvaccinated mothers exhibited significant histopathological lesions, suggesting that vaccination offers a protective effect on the placenta and reduces the risk of SARS-CoV-2 placentitis and stillbirth (61). In a separate study, chickens were immunized with the Razi Clone12IR vaccine and subsequently challenged with *Newcastle disease* (ND). Histopathological examinations of various tissue and organ sections, including the trachea, lungs, cecal tonsils, spleen, bursa of Fabricius, liver, and small intestine, were conducted. The results demonstrated that the Razi Clone12IR vaccine could preserve the integrity of tissue and organ structures (62). Zhang et al. administered anti-*Streptococcus agalactiae*-specific IgY antibodies to tilapia for a duration of 10 days, followed by a challenge with *S. agalactiae*. Histopathological analyses revealed that the specific IgY effectively reduced tissue damage and preserved the integrity of tissue structure (55). In this study, *C. auratus* were immunized with the protein active vaccine, DNA vaccine, and IgY antibody vaccine of VF17320. Following a viral challenge, histopathological examinations were conducted on the kidneys, spleen, and intestines of the *C. auratus*. The results indicated that the morphological structures of the kidneys, spleen, and intestines in the experimental group remained intact, with no evidence of apoptotic damage. These findings suggest that the three vaccines targeting VF17320 can protect the morphological integrity of internal organs.

The immunofluorescence method can detect the expression of p53 and γH2A.X, thereby serving as a tool for drug function identification. Zhang et al. demonstrated through immunofluorescence experiments that specific IgY could decrease apoptosis in intestinal epithelial cells and reduced caspase activity. This suggests that specific IgY may be a potential vaccine candidate for gastrointestinal pathogen infections (55). Liu et al. immunized *C. auratus* with IgY antibodies targeting the outer membrane proteins of *P. fluorescens* (PF1380 and ExbB) and subsequently challenged the fish with the pathogen. The presence of IgY antibodies was confirmed through pathological sections and protein immunofluorescence analyses of p53 and γH2A.X. Furthermore, the outer membrane proteins OmpW and Slp were shown to protect the structural and functional integrity of visceral tissues (27). In this study, the VF17320 protein active vaccine, DNA vaccine, and IgY antibody vaccine were utilized to immunize *C. auratus*. The apoptosis factor p53 and the DNA damage factor γH2A.X were assessed, and the average fluorescence intensity of p53 and γH2A.X showed a significant decrease, which indicated that the three vaccines can mitigate visceral DNA damage and cell apoptosis in *C. auratus*.

This study investigated the immunoprotection of protein, DNA, and IgY antibody vaccines of VF17320 protein. Both protein and DNA vaccines belong to active immunization vaccines. Beyond the structural differences between the protein and DNA constructs, the disparity in immune protection may be attributed to differences in antigen processing, memory cell generation, and duration of immune activation (63). DNA vaccines is the process of constructing target gene into plasmid, and then the recombinant plasmid is immunized into animal cells to transcribe and express the corresponding protein. It is known to elicit both humoral and cellular immunity and may promote longer-lasting memory responses due to endogenous antigen expression (64). In contrast, protein vaccines transcribe and express target genes in an exogenous host through gene cloning method, and obtain corresponding proteins using protein purification techniques, and then immunize the animal to activate the immune response. Protein vaccines mainly activate humoral immunity and have weaker induction of cellular immunity, and typically elicit a more immediate but possibly shorter-lived immune response (13). Additionally, protein immunity requires to mix with adjuvants (aluminum hydroxide, Freund’s adjuvant, mineral oil) to enhance the immune activity of proteins; DNA vaccines usually do not require adjuvants and are easy to immunize. In this study, the DNA and protein vaccines of VF17320 activated the immune response of fish, but DNA vaccine is relatively convenient for fish immunization. IgY antibody belongs to passive immunization vaccine, and can confer immediate protection and may be especially effective in the early stages of acute infection or when the host’s immune system is naive or compromised (15). While active immunization requires time to develop an adaptive immune response, IgY administration can neutralize pathogens directly upon exposure (65). Some researchers have shown that IgY passively immunized animals, and its immune effect against bacteria can even be produced after 15 min. Our research shows that the immune activity of IgY is better on 2 h after immunization and can last for more than 10 days in the animal body. Overall, this study prepared protein, DNA, and IgY antibody vaccines of VF17320 with high immune activity, providing a reference for the prevention and control of major pathogens in aquaculture.

## 5 Conclusion

In conclusion, this study evaluated the efficacy of the protein, DNA and IgY antibody vaccines of VF17320 in *C. auratus* against challenges posed by *V. fluvialis* and *A. hydrophila*. The results demonstrated that the three vaccines significantly enhanced protection against bacterial infections, reduced bacterial counts in the kidneys and improved the phagocytic activity of leukocyte in both chickens and *C. auratus*. *In vitro* experiments revealed that *C. auratus* serum effectively recognized the two pathogenic bacteria, with the IgY antibody titer for VF17320 reaching 1: 25000. Furthermore, the vaccines increased the expression of immune factors in *C. auratus* and enhanced their anti-inflammatory and antioxidant activities. Moreover, these vaccines also preserved the integrity of visceral tissues, and decreased both apoptosis and DNA damage in visceral tissue cells in *C. auratus*. Collectively, the protein, DNA and IgY antibody vaccines of VF17320 exhibit immune protective activities against various bacterial infections, indicating their potential as polyvalent vaccine candidates for fish.

## Data Availability

The original contributions presented in the study are included in the article/Supplementary material, further inquiries can be directed to the corresponding authors.
