# Supplementary material for: Evaluation of the multivalent immune protective effects of the Vibrio fluvialis outer membrane protein VF17320, and its DNA and IgY antibody vaccines in fish
Source: Front Vet Sci. 2025 Jun 18;12:1586258. doi: 10.3389/fvets.2025.1586258 (PMC12213336; doi:10.3389/fvets.2025.1586258)
Supplement: Supplementary file 1 [file Data_Sheet_1.zip › Supplementary Files/SUPPLEMENTARY FIGURE 2.pdf]

A B C

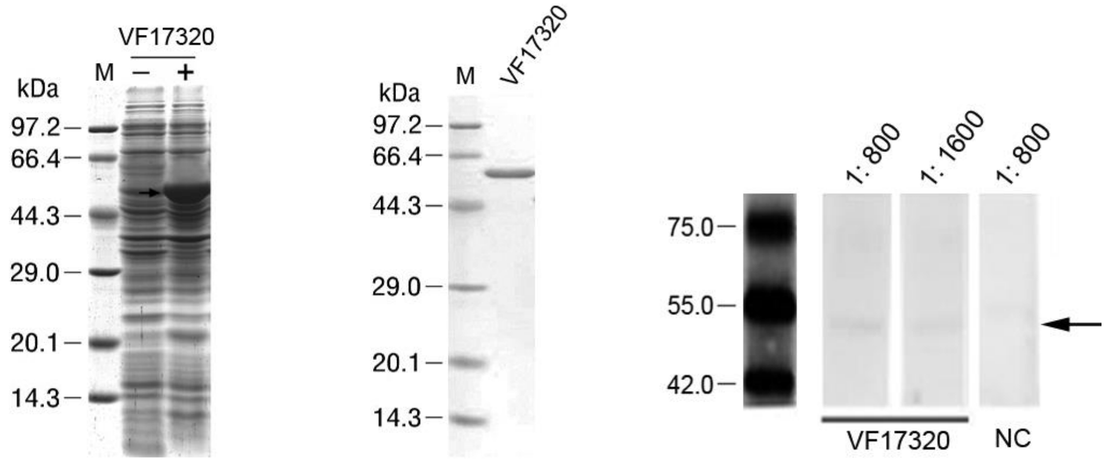

SUPPLEMENTARY FIGURE 2

The expression, purification, and verify of VF17320 protein. (A) The expression of VF17320 with SDS-PAGE. (B) The purification of VF17320 with SDS-PAGE. (C) The verify of VF17320 with western blotting. (–) Uninduced VF17320 strain with IPTG. (+) Induced VF17320 strain with IPTG.
