# Supplementary material for: Evaluation of the multivalent immune protective effects of the Vibrio fluvialis outer membrane protein VF17320, and its DNA and IgY antibody vaccines in fish
Source: Front Vet Sci. 2025 Jun 18;12:1586258. doi: 10.3389/fvets.2025.1586258 (PMC12213336; doi:10.3389/fvets.2025.1586258)
Supplement: Supplementary file 1 [file Data_Sheet_1.zip › Supplementary Files/SUPPLEMENTARY TABLE 1.pdf]

SUPPLEMENTARY TABLE 1 Primers used for the qRT-PCR.

Gene	NCBI number	Forward primer (5–3’)	Reverse primer (5–3’)
il-6	XM_026289280.1	TCTCCTCAGACCCTCAGACG	CGTTTGGTCCCGTGTTTGAC
il-8	XM_026267284.1	GGAGTGCAGGCCACTGTTAG	ATCAGAAGCATGAAGGCGGA
il-1β	AJ249136.1	TTCAGGAAAGAGACGGGCAC	GTCAGTTGGCACCTGGATCA
tnf-α	EU069817.1	GGGCCACATCGTGATTCGTA	GCCTCCAGTGTAGCATGTGT
gapdh	XM_026284269.1	GATTTCAACGGGGATGTGCG	TCACACACACGGTTGCTGTA
