# Supplementary figures and images for: Evaluation of the multivalent immune protective effects of the Vibrio fluvialis outer membrane protein VF17320, and its DNA and IgY antibody vaccines in fish
Source: Front Vet Sci. 2025 Jun 18;12:1586258. doi: 10.3389/fvets.2025.1586258 (PMC12213336; doi:10.3389/fvets.2025.1586258)

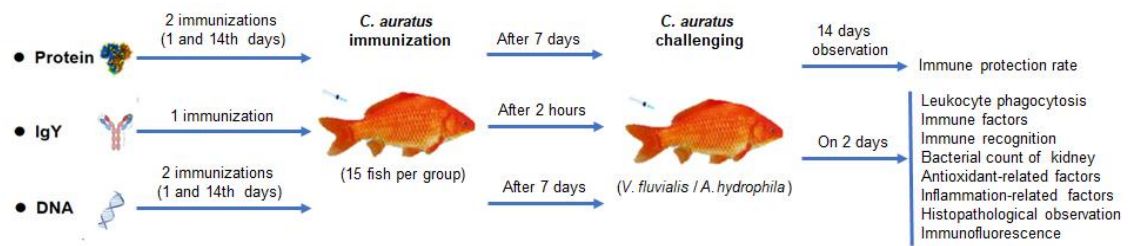

**SUPPLEMENTARY FIGURE 1**  
Experimental process.

Supplement: Supplementary file 1 [file Data_Sheet_1.zip › Supplementary Files/SUPPLEMENTARY FIGURE 1.pdf]
